# The Mesoscopic Modeling of Burst Suppression during Anesthesia

**DOI:** 10.3389/fncom.2013.00046

**Published:** 2013-04-30

**Authors:** David T. J. Liley, Matthew Walsh

**Affiliations:** ^1^Brain and Psychological Sciences Research Centre, Faculty of Life and Social Sciences, Swinburne University of TechnologyHawthorn, VIC, Australia

**Keywords:** burst suppression, anesthesia, electroencephalogram, mean field model, neuronal hyperexcitability

## Abstract

The burst-suppression pattern is well recognized as a distinct feature of the mammalian electroencephalogram (EEG) waveform. Consisting of alternating periods of high amplitude oscillatory and isoelectric activity, it can be induced in health by deep anesthesia as well as being evoked by a range of pathophysiological processes that include coma and anoxia. While the electroencephalographic phenomenon and clinical implications of burst suppression have been studied extensively, the physiological mechanisms underlying its emergence remain unresolved and obscure. Because electroencephalographic bursting phenomenologically resembles the bursting observed in single neurons, it would be reasonable to assume that the theoretical insights developed to understand bursting at the cellular (“microscopic”) level would enable insights into the dynamical genesis of bursting at the level of the whole brain (“macroscopic”). In general action potential bursting is the result of the interplay of two time scales: a fast time scale responsible for spiking, and a slow time scale that modulates such activity. We therefore hypothesize that such fast-slow systems dynamically underpin electroencephalographic bursting. Here we show that a well-known mean field dynamical model of the electroencephalogram, the Liley model, while unable to produce burst suppression unmodified, is able to give rise to a wide variety of burst-like activity by the addition of one or more slow systems modulating model parameters speculated to be major “targets” for anesthetic action. The development of a physiologically plausible theoretical framework to account for burst suppression will lead to a more complete physiological understanding of the EEG and the mechanisms that serve to modify ongoing brain activity necessary for purposeful behavior and consciousness.

## 1 Introduction

**Figure 1:**
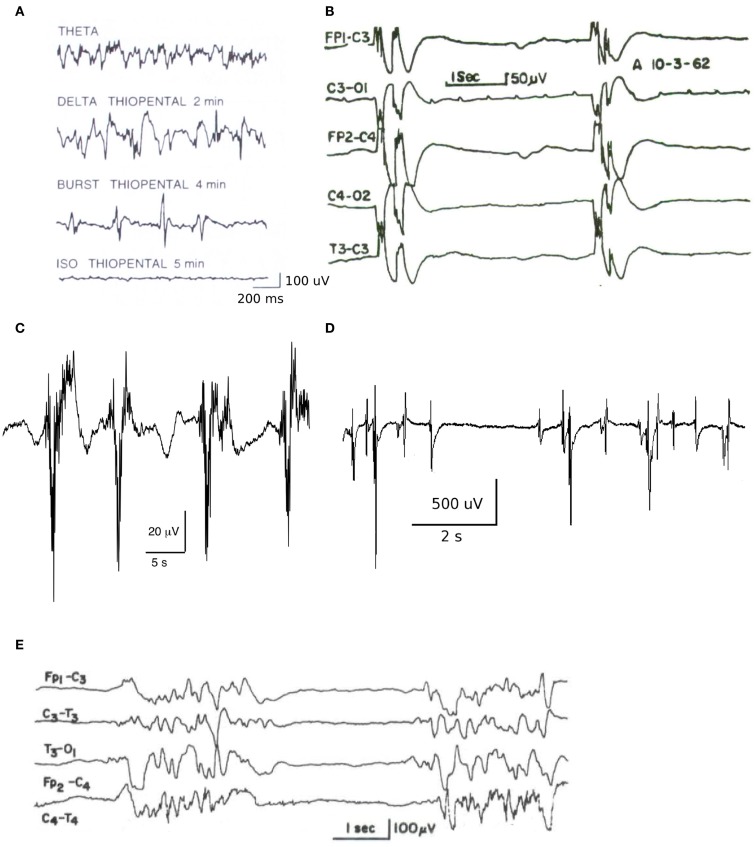
**Example traces of electroencephalogram and electrocorticogram illustrating the heterogeneity of BS patterns**. **(A)** Changes in neocortical electroencephalogram in the rat, recorded using dural surface electrodes, in response to a 5 mg/kg/min thiopental infusion [figure reproduced with permission from Lukatch and MacIver (1996)]. **(B)** Electroencephalogram recorded in acute anoxia showing a clear burst-suppression pattern with grouped spikes [figure reproduced with permission from Hockaday et al. (1965)]. **(C)** BS pattern during closed loop target controlled propofol infusion at a target level of approximately 15 μg/ml (data courtesy of Professor Michel Struys, Groningen). Note the bursts consist of fast activity (>10 Hz) on a slow wave background. **(D)** Electrocorticogram obtained from an adult merino sheep during deep enflurane anesthesia, demonstrating high amplitude spikes interspersed with isoelectric periods of variable length [figure reproduced with permission from Voss et al. (2006)]. **(E)** Electroencephalogram recorded from a 3-month-old infant suffering from infantile myoclonic encephalopathy [reproduced with permission from Niedermeyer (2005)].

Prior to the development of the modern intensive care unit in the early 1960s, that featured intubation, artificial respiration, and comprehensive physiological monitoring, reports of the electroencephalographic pattern of burst suppression (BS) were confined to animal studies involving deep anesthesia and the occasional case of psychosurgery (Niedermeyer, 2009). Since then the burst-suppression pattern has become well recognized as a major diagnostic feature of the EEG waveform that is encountered in a range of encephalopathic conditions, in addition to its appearance in health during deep anesthesia. Typically the BS pattern consists of bursts of high amplitude slow, sharp, or spiking electroencephalographic activity separated by periods of electroencephalographic suppression (isoelectricity). The oscillatory features of the bursts, together with their duration and the duration of suppressed periods show a high degree of variability (see Figure 1 for examples) that presumably reflects its myriad of initiating causes. First identified during deep anesthesia with tribromoethanol in cats (Derbyshire et al., 1936), labeled burst-suppression pattern by Swank and Watson (1949) during barbiturate and ether anesthesia in dogs, it is now associated with cortical deafferentation (Henry and Scoville, 1952), cerebral anoxia and hypoxia, various types of intracortical lesions (Fischer-Williams and Cooper, 1963), deep coma, various infantile encephalopathies, the final stages of deteriorated status epilepticus (Treiman et al., 1990), hypothermia, and high levels of many sedative and anesthetic agents (Schwartz et al., 1989; Akrawi et al., 1996).

Burst suppression in the absence of anesthesia is in general associated with a very poor prognosis. For example in neonates (Grigg-Damberger et al., 1989) the appearance of BS, even if transient, is a portent of death or severe neurodevelopmental disability. In contrast, in adult populations while an anoxic/hypoxic BS pattern signals a serious pathophysiological event the outcome is not necessarily fatal and recovery with or without severe neurological damage is possible (Niedermeyer, 2009). Consistent with this are results of experimental work with EEG monitoring in rats revealing that animals with greater rates of high amplitude bursts have a better survival and neurological outcome compared to those with lower rates of low amplitude bursts (Geocadin et al., 2002).

While the electroencephalographic phenomenon and clinical implications of BS have been studied extensively (Brenner, 1985; Niedermeyer, 2009) the physiological mechanisms underlying its emergence remain in general unresolved and obscure.

Burst suppression is typically thought to be spatially homogeneous with burst onset and termination reported to occur near simultaneously across the entire scalp (Brenner, 1985; An et al., 1996; Ching et al., 2012), indicating that low level subcortical mechanisms may be playing a decisive role. However arguing against this is the fact that this pattern persists subsequent to cortical deafferentation (Lukatch and MacIver, 1996), indicating that it probably represents an intrinsic, though physiologically abnormal, dynamical mode of cortex. Indeed the phenomenal resemblance of the patterns of BS to disorders of neuronal hyperexcitability suggests the involvement of similar physiological mechanisms. For example the bursting during burst suppression is often associated with myoclonic jerks resembling those seen during epileptic fits. Like generalized epileptiform activity, bursts are recorded simultaneously at multiple electrode derivations, implying the wide synchronization of neuronal activity.

At the cellular level a commonly reported finding is that hyperpolarization of the membrane potential of cortical neurons reliably precedes any overt electroencephalographic activity of BS (Steriade et al., 1994). Such hyperpolarization, which has been attributed to an increase in neuronal membrane potassium conductance (Steriade et al., 1994), has been hypothesized to play a major role in the induction of BS. This implied importance of inhibition in the genesis of BS is further supported by results involving rat neocortical brain slice micro-EEG preparations in which the application of a direct acting GABA*_A_* agonist, muscimol, readily induces BS. However contradicting this result is work reporting that inhibition is diminished during isoflurane-induced BS, in an *in vivo* feline preparation, as evidenced by increases in cortical neuronal input resistance and extracellular chloride concentration (Ferron et al., 2009). Of course it may be that slow periodic modulations in inhibition, rather than singular increases or decreases in inhibition, underpin BS. In support of this view is the recent model of Ching et al. (2012), in which alterations in brain metabolism, due to the effects of hypoxia or anesthesia, parametrically regulate an activity dependent slow modulation of an adenosine triphosphate-gated potassium channel conductance to give rise to BS. However modulations in inhibitory activity alone may not be sufficient to account for BS and more consideration might need to be given to other mechanisms. For example Kroeger and Amzica (2007) present empirical evidence suggesting that modulations in excitatory synaptic efficiency, due to the progressive depletion of interstitial calcium during the periods of high amplitude electroencephalographic activity and its recovery during isoelectric periods, might account for BS. Consistent with this are reports involving laboratory slice preparations in which burst suppression induced by thiopental, propofol, and isoflurane is abolished by the application of glutamate receptor antagonists (Lukatch and MacIver, 1996). Whatever the pathophysiology of BS is it is reasonably clear that it is unlikely to be accounted for by a unitary physiological perturbation. That the physiological factors identified to date in BS all lead to a single well defined state suggests the possibility of an unifying dynamical mechanism. Thus the best hope for progress in understanding the phenomena of BS may be theoretical.

How might we theoretically approach BS? The well studied dynamical mechanisms of action potential bursting (Izhikevich, 2007) may be able to provide vital insights into the mechanisms of bursting in the EEG. In general the dynamical mechanisms underlying bursting can be divided into two broad classes (i) fast-slow bursters in which there is a clear separation of the underlying time scales, with a fast system responsible for the fast spiking, and a slow system its slow modulation, and (ii) “hedgehog” bursters (Izhikevich, 2000) in which there is no clear separation of time scales. In terms of developing a theory of BS the former might represent the preferable starting point as the little empirical evidence that is available (Ching et al., 2012), at least in humans, suggests that alphoid activity, indicative of normal resting EEG, is preserved during the bursts of BS. Thus a theoretical starting point to understanding BS might be to consider the slow modulation of a dynamical system developed to describe the resting EEG.

One such dynamical system is the mesoscopic electrocortical model of Liley et al. (Liley et al., 1999, 2002, 2011; Bojak and Liley, 2005; Frascoli et al., 2011). This model is capable of accounting for a range of resting electroencephalographic phenomena that includes the alpha rhythm (Liley et al., 2002), the modulation of resting activity by sedative and anesthetic action (Bojak and Liley, 2005) as well as the proconvulsant properties of the latter (Liley and Bojak, 2005), all within a physiologically plausible/admissible parameter space. This model is therefore well suited as a foundation from which to explore the physiological and dynamical genesis of BS. However, because in this model rhythmogenesis emerges from a strong coupling between cortical excitatory and inhibitory population activity, in its present form it has a restricted ability to exhibit BS through the parametric separation of time scales, either through the simulated actions of anesthetics or through other parametric routes. Here we show that BS can however emerge in this model by the addition of a slow system driven by one or more of the originally defined mean fields. We speculate that such a slow system represents a mathematical ansatz for the slow neuromodulation of activity by a variety of intracortical, inter-cortical, and subcortical systems that include thalamus and the ascending neurotransmitter modulatory systems.

## 2 Materials and Methods

### 2.1 Mesoscopic mean field modeling of electrocortical activity

The electroencephalogram and electrocorticogram arise out of the cooperative activity of many thousands of neurons. A single electroencephalographic electrode records the synaptically induced currents of well over a 100,000 neurons (Nunez and Srinivasan, 2005) and thus detailing each neurons contribution to this summed activity would appear superfluous. For this reason it is preferable instead to model the activity of populations of neurons. One general way of achieving this, in which known stochastic fluctuations can be included, is to dynamically evolve the probability distributions associated with the states of the neuronal ensemble. While in principle providing a rigorous way forward the formulation of such stochastic equations of motion entails a great deal of physiological uncertainty. For this and other reasons (Deco et al., 2008) a more resolute path is to dynamically evolve some average quantity such as the mean soma membrane potential or the mean firing rate of some suitably defined neuronal ensemble. In this manner a mesoscopic level model can be developed which acts as a bridge between cellular (or microscopic) level activity and whole brain (or macroscopic) level behavior. While the current mathematical approach for formulating the equations of motion for the activity of neuronal populations or masses, stems principally from the works of Wilson and Cowan (1972, 1973), Nunez (1974), Freeman (1975), and Amari (1975), they are not particularly successful in articulating the genesis of rhythmic activity in the EEG and its modulation by pharmacological agents, due to a range of mathematical simplifications that cannot be justified by an appeal to physiology. For this reason a range of biologically more refined neuronal populations models have been developed (Deco et al., 2008) that form a more appropriate basis from which to model the physiological genesis of the electroencephalogram. The model of Liley et al. (Liley et al., 1999, 2002, 2011; Bojak and Liley, 2005; Frascoli et al., 2011) is one such model and will be the focus of our attempts to account for the phenomenon of BS.

**Figure 2:**
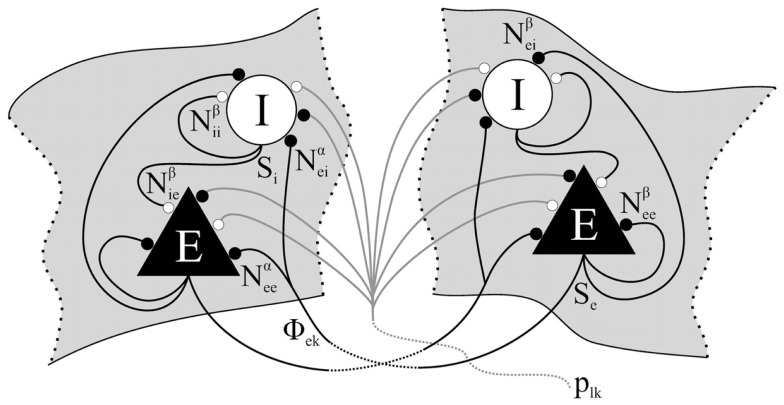
**Schematic overview of the essential intracortical and cortico-cortical interactions between excitatory and inhibitory neuronal populations in the model of Liley et al. (Liley et al., 1999, 2002, 2011; Bojak and Liley, 2005; Frascoli et al., 2011)**. Following conductance based approaches typically used to model networks of synaptically interacting networks of individual model neurons, excitatory and inhibitory neuronal populations are modeled as single passive resistive-capacitive compartments into which all synaptically induced postsynaptic currents flow. Functionally these populations are equivalent to the excitatory and inhibitory KO sets of Freemans K-set hierarchy (Freeman, 1975). Cortical activity is then described by the mean soma membrane potentials of the spatially distributed excitatory (*h_e_*) and inhibitory (*h_i_*) neuronal populations. The connection with physiological measurement is obtained through *h_e_*, which is assumed to be linearly related to the surface recorded electroencephalogram (Freeman, 1975; Nunez and Srinivasan, 2005). Figure reproduced with permission from Frascoli et al. (2011).

The model of Liley et al. is able to account for the major features of the mammalian electroencephalogram in the context of a parameterization that accords with physiological measurement and experiment. Because it aims to provide a dynamical description of the electroencephalogram the model is spatially coarse-grained over the approximate extent of a cortical macrocolumn, reflecting the general physiological wisdom that such columniation signifies populations of neurons having similar functional behavior and anatomical connectivity. The multiple interactions between individual neurons are replaced by effective feed-forward and feed-back interactions between the mean activity (or mean fields) of excitatory and inhibitory populations of neurons over the spatial extent of the column. In this way mammalian neocortex is conceived as consisting of localized populations of excitatory and inhibitory neurons interacting by all possible combinations of feed-forward and feed-back connectivity interacting globally via long-range excitatory connections (Figure 2).

Thus the response of the mean soma membrane potential *h_k_* (*k* = *e, i*) at position *r* on a two-dimensional cortical sheet, in response to induced post synaptic activity *I_lk_* (*l* = source, *k* = target population) is given by

(1) $$\tau_{k}\frac{\partial h_{k}\left(r,t\right)}{\partial t}=h_{k}^{r}-h_{k}\left(r,t\right)+\underset{l=e,i}{\sum }\frac{h_{lk}^{eq}-h_{k}\left(r,t\right)}{\left|h_{lk}^{eq}-h_{k}^{r}\right|}I_{lk}\left(r,t\right)$$

The postsynaptic response to a single pre-synaptic action potential (at *t* = 0) is modeled by the well-known synaptic alpha function of cable theory as Γ*_lk_*γ*_lk_t* exp(1 − γ*_lk_t*)Θ(*t*) where Γ*_lk_* is peak amplitude (occurring at *t* = *t*_peak_ = 1/γ*_lk_*) of the respective excitatory (*l* = *e*) or inhibitory (*l* = *i*) postsynaptic potential (PSP), and Θ(*t*) is the Heaviside step function. Thus we assume that the time course of the synaptically induced excitatory and inhibitory currents is described by a critically damped oscillator driven respectively by the mean rate of incoming excitatory and inhibitory axonal pulses:

(2) $${\left(\frac{\partial }{\partial t}+\gamma_{lk}\right)}^{2}I_{lk}\left(r,t\right)=\exp\left(1\right)\Gamma_{lk}\gamma_{lk}A_{lk}\left(r,t\right),$$

with

(3) $$\begin{array}{lll} A_{ek}\left(r,t\right) & =N_{ek}^{\beta }S_{e}\left[h_{e}\left(r,t\right)\right]+\phi_{ek}+p_{ek}\left(r,t\right), & \end{array}$$

and

(4) $$\begin{array}{llll} A_{ik}\left(r,t\right) & =N_{ik}^{\beta }S_{i}\left[h_{i}\left(r,t\right)\right]+p_{ik}\left(r,t\right), & & \end{array}$$

where *A_lk_* comprises the different sources of pre-synaptic spikes: $N_{lk}^{\beta }S_{i}$ (input from local cortical neuronal populations), *Φ_ek_* (input from long-range excitatory cortico-cortical fibers), and *p_lk_* (extra-cortical sources). While the present consensus is that extra-cortical sources (thalamo-cortical afferents) are purely excitatory in nature and thus *p_ik_* = 0, we choose to retain these terms as when time independent they can be utilized to include the effects of tonic inhibition that are known to be induced by anesthetic action. The time courses of the synaptically induced currents, 1/γ*_lk_* are taken to describe the time course of “fast” excitatory [*l* = *e*: α-amino-3-hydroxl-5-methyl-4-isoxazole-propionate (AMPA) and kainate] and inhibitory [*l* = *i*: γ-amino-butyric-acid type A (GABA_A_)] neurotransmitter kinetics. Thus each type of PSP (excitatory, inhibitory) is described by two parameters Γ*_lk_*, γ*_lk_*. However, as we will describe later, a parametrically more flexible description of the PSP is required to meaningfully model the effects of anesthetics in which we can independently vary peak amplitude, rise (*t*_peak_) and decay times. Mean neuronal population firing rates, *S_l_*, are assumed to instantaneous sigmoid functions of the mean soma membrane potential i.e.,

(5) $$S_{l}\left[h_{l}\left(r,t\right)\right]=S_{l}^{\max}∕\left\{1+\exp\left[\sqrt{2}\left(h_{l}\left(r,t\right)-{\bar{\mu }}_{l}∕\sigma_{l}\right)\right]\right\}$$

The axonal pulses *Φ_ek_* propagated by the exclusively excitatory long-range cortico-cortical fiber system is in the simplest case described by the following two-dimensional telegraph equation,

(6) $$\begin{array}{llll} & {\left(\frac{\partial }{\partial t}+v_{ek}\Lambda_{ek}\right)}^{2}\phi_{ek}\left(r,t\right)-\frac{3}{2}v_{ek}^{2}\nabla^{2}\phi_{ek}\left(r,t\right) & & \\ & \;=v_{ek}^{2}\Lambda_{ek}^{2}N_{ek}^{\alpha }S_{e}\left[h_{e}\left(r,t\right)\right] & \end{array}$$

where $N_{ek}^{\alpha }$ is the total number of excitatory connections formed by long-range cortico-cortical axons on long on local population *k*, and assumes a single axonal conduction velocity *v_ek_* and an exponential fall off with distance (characteristic scale = 1/Λ*_ek_*) of the strength of cortico-cortical connectivity. For simplicity, and given the fact that at least in anesthesia BS appears to have a degree of spatial uniformity, we chose to only study the spatially homogeneous case, i.e., ▽^2^ = 0.

Equations (1)–(4) and (6) represent a system of 8 coupled non-linear partial differential equations that typically defines the Liley model of electrocortical rhythmogenesis, which is capable of reproducing the main features of spontaneous human electroencephalogram (alpha resonance, “1/*f*” activity). Table 1 summarizes all model parameters, their definitions, and approximate ranges.

**Table 1:** **List of spatially averaged parameters for different types *k*, *l* ∈ {*e*, *i*} of neuronal target populations in the electrocortical model of Liley et al. (Liley et al., 1999, 2002, 2011; Bojak and Liley, 2005; Frascoli et al., 2011), with typical ranges that are assumed to be physiologically admissible**.

	Definition	Min	Max	Units
$h_{k}^{r}$	Resting membrane potential	−80	−60	mV
*τ_k_*	Passive membrane decay time	5	150	ms
$h_{ek}^{eq}$	Excitatory reversal potential	−20	10	mV
$h_{ik}^{eq}$	Inhibitory reversal potential	−90	$h_{k}^{r}-5$	mV
Γ*_ek_*	EPSP peak amplitude	0.1	2.0	mV
Γ*_ik_*	IPSP peak amplitude	0.1	2.0	mV
1/γ*_ek_*	EPSP rise time to peak	1	10	ms
1/γ*_ik_*	IPSP rise time to peak	2	100	ms
$N_{ek}^{\alpha }$	Number of excitatory cortico-cortical synapses	1000	5000	–
$N_{ek}^{\beta }$	Number of excitatory intracortical synapses	2000	5000	–
$N_{ik}^{\beta }$	Number of inhibitory intracortical synapses	100	1000	–
*v_ek_*	Axonal conduction velocity	0.1	1	mm ms^−1^
1/Λ*_ek_*	Decay scale cortico-cortical connectivity	10	100	mm
$S_{k}^{\text{max}}$	Maximum firing rate	0.05	0.5	ms^−1^
$\bar{\mu }k$	Mean firing threshold	−55	−40	mV
*σ_k_*	Firing threshold standard deviation	2	7	mV
*p_lk_*	Extra-cortical synaptic input rate	0	10	ms^−1^

*Table adapted from Liley et al. (2011). EPSP, excitatory PSP; IPSP, inhibitory PSP*.

### 2.2 Model parameterization: Generation of normative parameter sets

Because BS activity (at least that induced by anesthetic and sedative action) is assumed to ultimately arise out of a background of normal electroencephalographic activity it is important to define parametrically normative states in order to study how they may be perturbed during health and disease. We therefore chose to utilize previously defined parameter sets (Bojak and Liley, 2005) that have the following properties: (i) are confined to the physiologically admissible parameter space (see Table 1), (ii) give rise to electroencephalographically and physiologically plausible activity (“1/*f*” decay at low frequencies plus a relatively sharp peak at alpha frequencies, 8–13 Hz; mean excitatory/inhibitory neuronal firing rates <20 s^−1^) and (iii) that exhibit transient increases in total power and monotonic reductions in mean frequency with respect to modeled anesthetic action (see below). In general such sets can be found by randomly searching the high dimensional physiologically admissible (and plausible) parameter space. For further details see Bojak and Liley (2005).

### 2.3 Modeling anesthetic action

The range of molecular and cellular targets identified to date as sites of anesthetic action is so varied that a unitary biological mechanism for anesthetic effect seems unlikely. Nevertheless, at least functionally, at the level of cortex anesthetics seem to act principally by enhancing the actions of inhibitory activity (Liley et al., 2011). Indeed from the perspective of the mean field model we have described many of its parameters can be related in a fairly straightforward way to these alterations in inhibitory activity and other identified sites of anesthetic action in cortex (see Table 2). However a parametrically more flexible description of the PSP, than is presently incorporated, is required to meaningfully model the effects of anesthetics in which we can independently vary peak amplitude, rise (*t*_peak_) and decay times. For example isoflurane, a volatile halogenated anesthetic, has been shown to prolong the decay time of the unitary IPSP without altering its time to peak. Fortunately a simple modification of the equation describing the dynamics of the PSP enables independent adjustment of the peak amplitude, rise (*t*_peak_) and decay times. By defining *I_lk_* to satisfy

(7) $$\begin{array}{llll} & \left[\frac{\partial }{\partial t}+\gamma_{lk}\left(\varepsilon_{lk}\right)\right]\left[\frac{\partial }{\partial t}+{\tilde{\gamma }}_{lk}\left(\varepsilon_{lk}\right)\right]I_{lk}\left(r,t\right) & & \\ & \;={\tilde{\gamma }}_{lk}\left(\varepsilon_{lk}\right)\exp\left[\gamma_{lk}\left(\varepsilon_{lk}\right)∕\gamma_{lk}^{0}\right]\Gamma_{lk}A_{lk}\left(r,t\right), & \end{array}$$

(8) $$\gamma_{lk}\left(\varepsilon_{lk}\right)=\varepsilon_{lk}\gamma_{lk}^{0}∕\left(e^{\varepsilon_{lk}}-1\right),\;\;{\tilde{\gamma }}_{lk}\;=\;\gamma_{lk}\left(\varepsilon \right)e^{\varepsilon_{lk}}$$

where 1/$\gamma_{lk}^{0}$ defines the time to peak, we can control the decay of the unitary PSP by altering *ε_lk_* > 0. Increasing *ε_lk_* will monotonically increase the decay time of the tail of the unitary PSP (see lower left panel, Figure 6). Empirically it is found that increasing the aqueous concentration of a range of GABAergic anesthetic agents leads to a progressive increase in the decay time of the unitary inhibitory PSP (e.g., Banks and Pearce, 1999) and thus *ε_ik_* will be a monotonic function of anesthetic concentration *c*, i.e., *ε_ik_*(*c*). Liley et al. (2011), based on a range of empirical evidence, have numerically estimated *ε_ik_*(*c*) for the volatile anesthetic agent isoflurane. However because *ε_ik_*(*c*) is not currently known for other GABAergic anesthetic agents we will assume that in general *ε_ik_* ∝ *c*.

**Table 2:** **Relationship between major experimentally identified sites of cortical anesthetic action and parameters of the electrocortical model of Liley et al. (Liley et al., 1999, 2002; Bojak and Liley, 2005)**.

Site of action	Main anesthetic effect	Parameters
2PK channels and extrasynaptic GABA_A_	Increase in tonic inhibition	*p_ik_*, $h_{k}^{r}$
nACh receptors	Reduction in tonic excitation	*p_ek_*, $h_{k}^{r}$
Synaptic GABA_A_	Increase of IPSPs	γ*_ik_*, Γ*_ik_*
AMPA/kainate receptors and NMDA receptors*	Reduction of EPSPs	γ*_ek_*, Γ*_ek_*
Myelinated axons	Slowdown of conduction^†^	*v_ek_*
Na channels	Alteration of neuronal firing	$S_{k}^{max}$, ${\bar{\mu }}_{k}$, *σ_k_*

*Parameters will depend on membrane potential in this case.

*^†^Effect demonstrated in periphery, speculative in cortex (Swindale, 2003)*.

It is worth noting that equation (7) reduces to equation (2) as *ε_lk_* → 0. Further details regarding this formulation can be found in Bojak and Liley (2005).

### 2.4 Defining a theoretical basis for bursting

We call a bursting system *fast-slow* if it can be written in the following form

(9) $$\begin{array}{lll} ẋ & =f\left(x,y\right)\;\;\;\;\;\left(fast\;oscillatory\;system\right) & \end{array}$$

(10) $$\begin{array}{lll} ẏ & =\mu g\left(x,y\right)\;\;\;\left(slow\;modulatory\;system\right) & \end{array}$$

where *x* ∈ ℝ*^m^* describes the m-dimensional system responsible for the fast oscillatory (spiking in single neuron models) dynamics and *y* ∈ ℝ*^n^* the n-dimensional slow system that modulates the fast oscillations (or spiking behavior). The parameter *μ* represents the ratio of the time scales between the slow and fast system. It is typically assumed that *μ* ≪ 1. Because *μ* can be made as small as we like equations (9) and (10) represent a singularly perturbed system.

We will assume that equations (1)–(4) and (6), which putatively describe the genesis of the “fast oscillatory” resting EEG, correspond to the m-dimensional fast system. To motivate the slow n-dimensional slow modulatory system we will make a plausible appeal to the biophysics of synaptic resource depletion and recovery during periods of sustained neuronal population activity. During periods of high firing neural activity a variety of factors come into play to diminish synaptic efficiency. The most important of these are receptor desensitization and synaptic vesicle depletion. Tsodyks and Markram (1997) developed a model to account for the biophysics of such activity dependent short term synaptic depression estimating that its onset is rapid, of the order of milliseconds, but that its recovery is quite slow, of the order of 800 ms. Given that such a time scale is approximately at least an order of magnitude greater than the characteristic time scales associated with resting EEG activity, this may represent a candidate slow EEG modulatory system. On this basis we choose to include this activity dependent short term synaptic depression using the following two different formulations, referred respectively to as SS1 and SS2,

(11) $$\begin{array}{lll} {\overset{{}^{\circ}}{\Gamma }}_{lk} & =\mu_{l}\left[\theta_{l}-k_{l}S_{l}\left(h_{l}\right)\right],\;\;\;\left(SS1\right) & \end{array}$$

(12) $$\begin{array}{lll} {\overset{{}^{\circ}}{\Gamma }}_{lk} & =\mu_{l}\left[\Gamma_{lk}^{0}∕\left(1+\exp\left[\kappa_{l}\left(h_{l}-\xi_{l}\right)\right]\right)-\Gamma_{lk}\right],\;\;\;\left(SS2\right) & \end{array}$$

where 1/*μ_l_* is the characteristic time scale of the respective slow modulatory system. Based on Tsodyks and Markram (1997) we will fix 1/*μ_l_* to 1000 ms. The advantage of the first formulation is that the rates of synaptic recovery (*μ_l_θ_l_*) and depletion (*μ_l_k_l_*) can be separately specified. The advantage of the second formulation is that Γ*_lk_* remains bounded between $\Gamma_{lk}^{0}$ (the resting value) and zero decreasing monotonically with increasing mean soma membrane potential *h_l_*, with $\Gamma_{lk}^{0}$ remaining as a free parameter. For low levels of the respective neuronal activity (*h_l_*) there is very little decrease in Γ*_lk_* until a threshold *ξ_l_* is reached, with the parameter *κ_l_* controlling the sensitivity of the change at this threshold to variations in neuronal activity. This formulation has previously been used by Tabak and Rinzel (2005) in their mean field model for spontaneous electrical bursting activity in embryonic chick spinal cord.

### 2.5 Computational methods

All numerical integrations and one-dimensional dynamical continuations were performed using the XPPAUT package (Ermentrout, 2002). A 4-th order Runge-Kutta scheme with a time step of 0.1 ms was used to numerically integrate the differential equations. Because of the multiple time scales our differential system may suffer from stiffness and numerical solutions may not converge. In these cases we have used the recommended “stiff” integrator CVODE (Cohen and Hindmarsh, 1994) as implemented in XPPAUT.

## 3 Results

**Figure 3:**
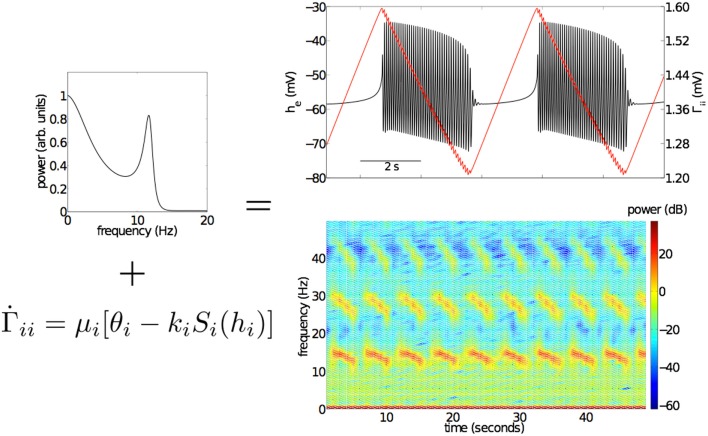
**Effects of the slow, activity dependent modulation of IPSPs on the inhibitory neuronal population, Γ*_ik_* using the SS1 model of equation (11)**. The top left panel shows the fluctuation spectrum of the unmodulated “fast” EEG system (Bojak and Liley, 2005). When Γ*_ik_* of this system is slowly modulated (*μ_i_* = 0.001 ms^−1^, *θ_i_* = 0.1818 mV, *k_i_* = 10 mV s) bursting emerges (top right panel). Bursting is associated with the periodic modulation of Γ*_ii_* (red line, top right panel) on a much longer time scale than that of intra-burst oscillatory activity. Time-frequency analysis (bottom right panel) reveals that intra-burst activity sweeps down through a range of frequencies from low beta (13–30 Hz) to high alpha (8–12 Hz). Parameter values used for the “fast” EEG system can be found in Table 3.

All numerical simulations were performed using a single model parameter set having a physiologically plausible white noise fluctuation spectrum (see top left panel Figure 3) and a single stable fixed point. This parameter set was found using the methods described in section 2.2. The parameters used, all within the physiologically admissible domain, can be found in the Table 3.

**Table 3:** **Model parameter set used in simulations of Figures 3–8**.

Parameter	Value	Parameter	Value
$h_{e}^{rest}$ (mV)	−68.1355	$N_{ee}^{\alpha }$	4994.4860
$h_{i}^{rest}$ (mV)	−77.2602	$N_{ei}^{\alpha }$	2222.9060
$h_{ee}^{eq}$ (mV)	−15.8527	$N_{ee}^{\beta }$	4582.0661
$h_{ei}^{eq}$ (mV)	7.4228	$N_{ei}^{\beta }$	4198.1829
$h_{ie}^{eq}$ (mV)	−85.9896	$N_{ie}^{\beta }$	989.5281
$h_{ii}^{eq}$ (mV)	−84.5363	$N_{ii}^{\beta }$	531.9419
*τ_e_* (ms)	138.3660	*v_ek_* (cm ms^−1^)	0.1714
*τ_i_* (ms)	89.3207	Λ*_ek_* (cm^−1^)	0.2433
Γ*_ee_* (mV)	0.3127	$S_{e}^{max}$ (ms−^1^)	0.2801
Γ*_ei_* (mV)	0.9426	$S_{i}^{max}$ (ms−^1^)	0.1228
Γ*_ie_* (mV)	0.4947	*μ_e_* (mV)	−47.1364
Γ*_ii_* (mV)	1.4122	*μ_i_* (mV)	−45.3751
γ*_ee_* (ms^−1^)	0.4393	*σ_e_* (mV)	2.6120
γ*_ei_* (ms^−1^)	0.2350	*σ_i_* (mV)	2.8294
γ*_ie_* (ms^−1^)	0.0791	*p_ee_* (ms^−1^)	3.6032
γ*_ii_* (ms^−1^)	0.0782	*p_ei_* (ms^−1^)	0.3639

*Parameter set taken from Bojak and Liley (2005). Because parameters were obtained as part of a numerical search their full precision had been detailed, however their sensitivity to perturbation is much less than the precision reported (Bojak and Liley, 2005)*.

Figure 3 shows the effects of the activity dependent modulation of Γ*_ii_* on simulated mean field EEG activity using SS1 [equation (11)]. Bursts emerge periodically, with intervening near isoelectric intervals, apparently driven by slow variations in Γ*_ii_* (red line, top right panel). A spectrogram of a sufficiently long simulated time series reveals that the frequency of the model EEG activity decreases from low beta (≅ 15 Hz) to high alpha (≅ 12 Hz) over the period of the bursts. Such intra-burst “chirping” is a common feature of many of the topologically identified single neuronal bursters (Izhikevich, 2007). Of interest are the multiple harmonics of this dominant oscillatory activity.

**Figure 4:**
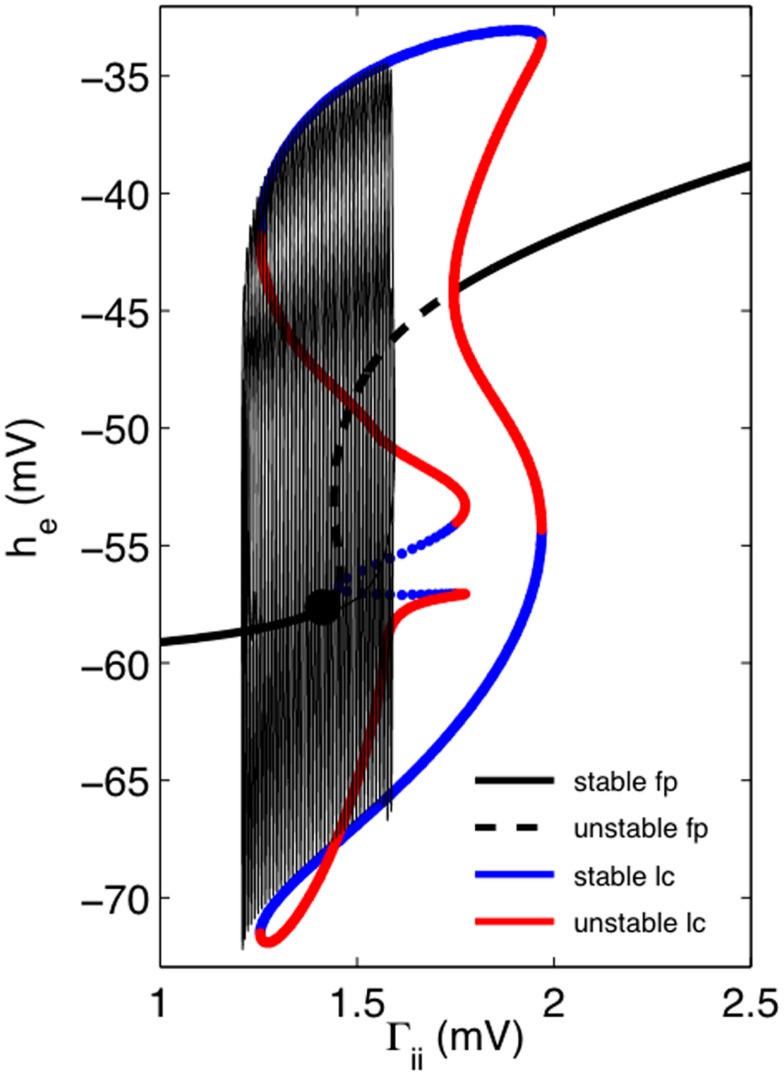
**One-dimensional continuation of the “fast” EEG system in *h_e_* suggests how we might account for the dynamics of bursting**. Thick black lines (solid = stable, dashed = unstable) shows the fixed points (fp) as a function of Γ*_ii_*. Solid black circle indicates fixed point for the default parameter values for “fast” EEG system (parameters as for Figure 3). Solid blue lines indicate the locus of maximum and minimum amplitudes respectively of stable limit cycle (lc) activity, whereas solid red lines correspond similarly to locus of unstable limit activity. Transitions between stable and unstable lc activity is predominantly through fold-limit cycle bifurcations, except for middle limit cycle branch where transition is through a torus bifurcation. Superimposed on this one-dimensional bifurcation diagram is the trajectory of a single burst.

Figure 4 illustrates how we might dynamically account for the transition to, and cessation from, bursting and follows the now standard method of the dissection of neural bursting pioneered by Rinzel (1985). Here we have set *μ_i_* = 0 and consider how the “fast” EEG system responds. Figure 4 shows a one-dimensional bifurcation diagram of this “fast” EEG system with Γ*_ii_* as the bifurcation parameter. Thick black lines show the fixed points as a function of Γ*_ii_*. For small values of Γ*_ii_* there is a single stable fixed point. As Γ*_ii_* is increased this fixed point loses stability by a super-critical Hopf bifurcation, thus signaling the onset of limit cycle activity. Periodic continuations of this low amplitude activity reveals that it, and a stable fixed point, co-exist with a higher amplitude limit cycle, thus suggesting that an activity dependent hysteresis drives the system between a fixed point and a high amplitude oscillation, thus giving rise to the bursting activity observed. This can be better seen by superimposing on this diagram the trajectory of a single burst (thin solid black line). Here we can see that the burst terminates through a fold-limit cycle bifurcation. At this stage it is not clear what bifurcation accounts for the emergence of the burst.

**Figure 5:**
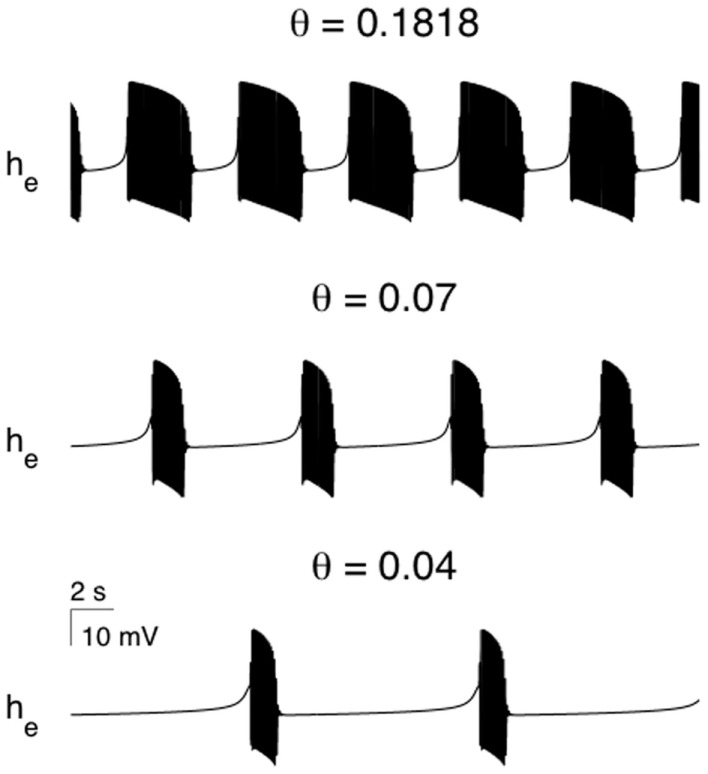
**The effect of parametrically varying the rate of recovery of synaptic efficacy in modulating the modeled burst duration and the quiescent (isoelectric) period**. If *θ_i_* is made small enough the bursting solutions will undergo a bifurcation to a non-oscillatory state. All parameters as per Figure 4.

A well described feature of anesthetic action is the reduction in cerebral blood flow and metabolism (Kaisti et al., 2003). Therefore during anesthetic action it would be reasonable to assume that the recovery of pre-synaptic neurotransmitter levels will be impaired. In particular as the anesthetic level increases then the rate of synaptic recovery should decrease. Figure 5 shows the effects of systematic reductions in the synaptic recovery rate for the SS1 model. As *θ_i_* (synaptic recovery) is decreased the burst duration decreases and the period of non-oscillatory isoelectric activity increases until the model EEG becomes fully isoelectric. This trend is also observed clinically during anesthesia.

**Figure 6:**
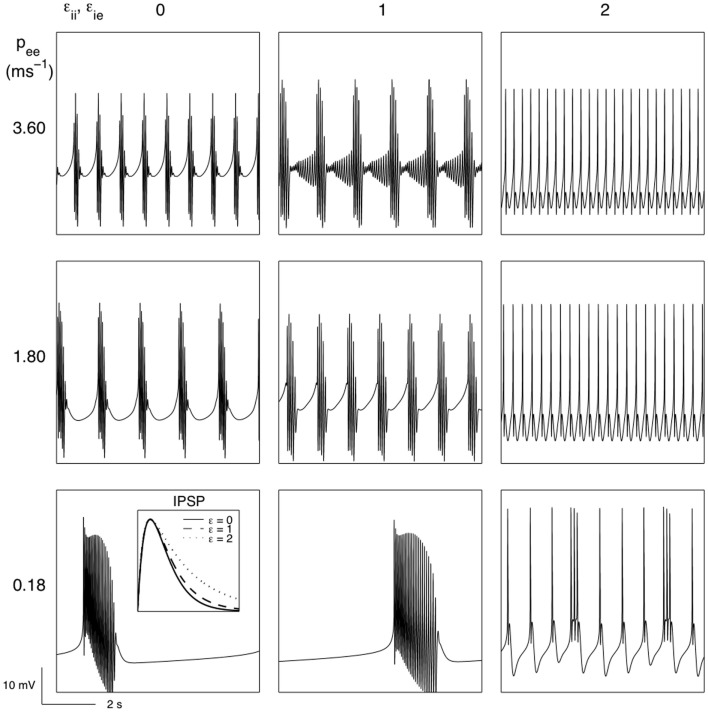
**The effects of short term synaptic depression of both excitatory and inhibitory cortical synapses in the genesis of burst suppression and its modulation by variations in extra-cortical input (*p_ee_*) and the IPSP decay time (*ε_ii_, ε_ie_*) (see section 2.3 for further details)**. Parameters: *θ_e_* = 0.1818 mV, *θ_i_* = 0.07 mV, *k_e_* = 14 mV s. All other parameters as for Figure 3.

Short term synaptic depression would be expected to affect all synapses, though the depression would not be expected to be uniform. So far we have assumed that the synaptic depression would principally affect inhibitory synapses between inhibitory neurons. Will such bursting survive when all types of “fast” synaptic activity is subject to the biological forces of short term synaptic depression? Figure 6 reveals that bursting does occur when both excitatory and inhibitory synaptic activity undergoes activity dependent short term synaptic depression. Further, the bursting that emerges is strongly modulated by parameters of the “fast” EEG system (see Table 2) that have been identified as targets for the action of anesthetic agents. Prolonging the decay of the unitary IPSP and reducing subcortical input are both found to significantly modulate modeled EEG bursting. In particular it is found that reducing excitatory extra-cortical input (*p_ee_*), which presumably dominantly arises from thalamus, leads to very long quiescent (isoelectric) periods.

**Figure 7:**
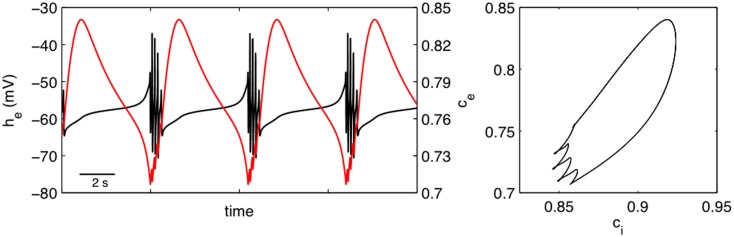
**Bursting produced by the activity dependent slow modulation of excitatory and inhibitory synaptic efficacy according to SS2 [equation (12)]**. Left panel shows *h_e_* (solid black line) and normalized excitatory synaptic efficacy *c_e_* ≡ Γ*_el_*/$\Gamma_{el}^{0}$ (solid red line) as a function of time. The left hand panel shows the phase relationship between normalized excitatory and inhibitory (*c_i_* ≡ Γ*_il_*/$\Gamma_{il}^{0}$) synaptic efficacies. Parameters: κ*_e_* = 0.2 mV^−1^, κ*_i_* = 0.1 mV^−1^, *ε_ii_* = 1.8, *ε_ie_* = 1.5.

One of the limitations in using SS1 is that the PSPs are, in principle, free to take any value whereas physiology would dictate that they should remain bounded. To explore the effects of this restriction we chose to define an alternative slow modulating system [SS2, equation (12)]. Figure 7 shows the effect of utilizing this system to provide a slow activity dependent modulation of excitatory and inhibitory synaptic efficacy of our “fast” EEG system. In the left panel of this figure we have plotted *c_e_* ≡ Γ*_el_*/$\Gamma_{el}^{0}$ as a function of time. The interesting thing to note is that in contrast to Figure 3 excitatory synaptic efficacy decreases during the quiescent interburst period and increases during the burst. The left hand panel however shows that there is a significant phase difference between the normalized excitatory synaptic efficacy *c_e_* ≡ Γ*_el_*/$\Gamma_{el}^{0}$ and the normalized inhibitory synaptic efficacy *c_i_* ≡ Γ*_il_*/$\Gamma_{il}^{0}$. This suggests that there is an important dynamical interplay between excitatory and inhibitory synaptic efficacy to regulate neuronal population excitability such that bursting occurs. This may explain why there is confusion in the empirical literature regarding the role alterations in synaptic efficiency have in the genesis of BS.

**Figure 8:**
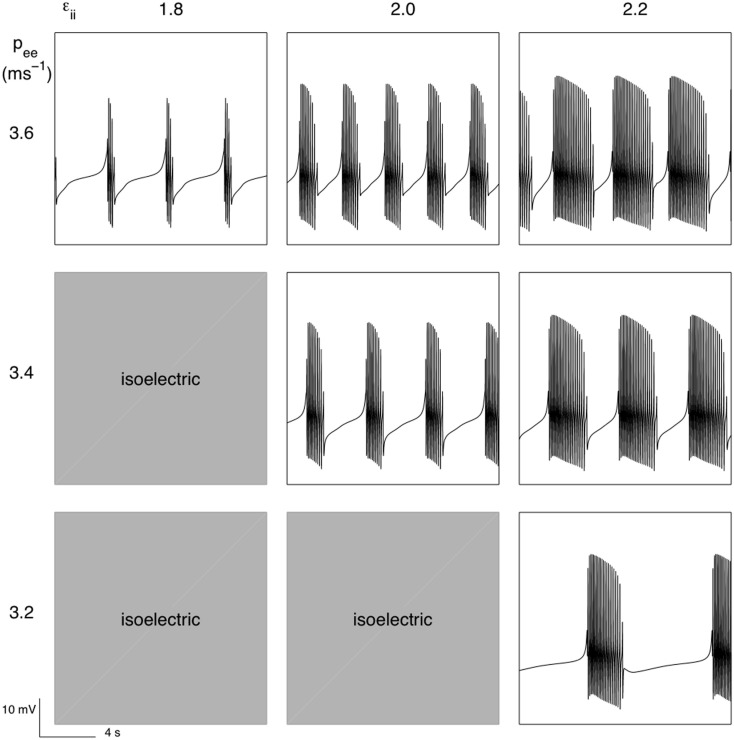
**Parametric variability of bursting produced by the activity dependent slow modulation of excitatory and inhibitory synaptic efficacy according to SS2 [equation (12)]**. For *ε_ii_* < 1.8 simulated EEG was isoelectric as was the case for labeled combinations of *ε_ii_* and *p_ee_*. Parameters as for Figure 7.

An important difference between SS2 and SS1 is that parameters hypothesized to be important targets of anesthetic action *p_ee_* and *ε_il_* are able not only to parametrically regulate bursting but appear also able to switch bursting on (presumably through a bifurcation from a stable fixed point). Figure 8 illustrates this. If the inhibitory neuronal IPSP decay time is not long enough then a single fixed point dominates which has an associated white noise fluctuation spectrum. But as the IPSP decay time increases (beyond *ε_ii_* > 1.8 for the parameter set chosen) then bursting emerges. However if *p_ee_* is decreased, as we would expect during anesthesia, then the isoelectric period is prolonged until at some critical value of *p_ee_* bursting is extinguished to be replaced by an infinitely long quiescent/isoelectric period.

## 4 Discussion

We have described here how a well-known model of the “fast” dynamics of the EEG can be modulated by a number of slow systems to produce bursting activity that bears some resemblance to BS seen clinically. The slow systems that we used were all based on some form of activity dependent short term plasticity that has been empirically observed, and used successfully in other models of macroscopic level bursting (Tabak and Rinzel, 2005). While we were able to clearly show the existence of bursting, because we did not include any additive or multiplicative noise sources, we were unable to account for the quasi-periodicity of BS. Thus all our busting arises from purely deterministic processes, presumably involving a range of well described bifurcations (Izhikevich, 2007). However because our system clearly exhibited bistability (see Figure 4) it is almost certain that our system will be able to exhibit some form of burst excitability in response to stochastic forcing. Such burst excitability has been described experimentally. For example during BS induced by various halogenated anesthetic agents, bursts can be readily evoked by auditory, visual, or somatosensory stimuli (Hartikainen et al., 1995). Nevertheless while burst onset and duration may be random variables it would seem that the bursts themselves should reveal a high degree of determinism (weak non-linearity) when compared to EEG in which bursting or epileptiform activity is not evident.

Because the parameter space of the underlying “fast” EEG model is potentially extremely large it is not possible to systematically explore its dynamical repertoire and it may be possible that this system, not augmented with one of the slow systems described, is able to burst. Nevertheless, on the basis of our results, and what is known regarding the dynamical mechanisms of bursting, it would seem likely that multiple pathways to BS exist through a variety of activity dependent slow modulatory systems.

**Figure 9:**
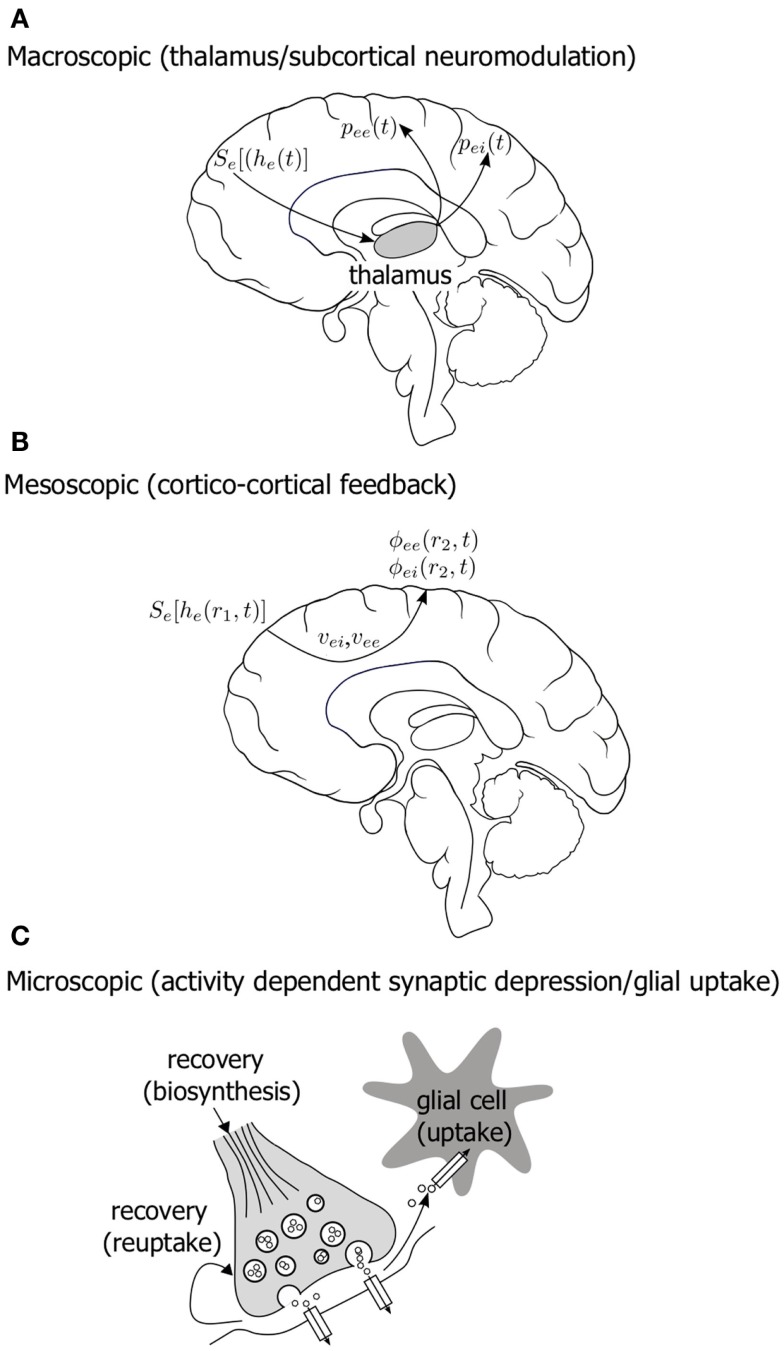
**Diagram illustrating a number of hypothesized, physiologically plausible, multiple scale slow modulatory systems that could be important in the genesis of electroencephalographic bursting in the context mean field model of Liley et al**. **(A)** Thalamo-cortical feed-back **(B)** changes in the conduction properties of the long-range cortico-cortical fiber system **(C)** slow changes in the efficacy of synaptic function due to activity dependent resource depletion and restitution. See accompanying text for symbol definitions.

Further we might hypothesize that such slow modulatory systems might span a number of functional scales in the brain. Figure 9 diagrammatically illustrates some possible candidate systems. An obvious activity driven slow modulatory system would be that associated with thalamus and the corresponding thalamo-cortical feed-back. Mean field models of the EEG that incorporate thalamo-cortical feed-back have been developed (Rennie et al., 2002) and it will be interesting to see if they are structurally configured to support BS. In addition to synaptic fatigue another obvious cortical system that might be marshaled to provide slow activity dependent modulation is the cortico-cortical conduction system. Although at this stage there is currently little evidence to suggest anesthetics slow conduction velocities, it is widely documented that axonal conduction velocities are significantly decreased in hypothermia. Decreases of up to 5% per °C for conduction velocity have been reported (Waxman, 1980). As BS has been observed to occur in hypothermia (Schwartz et al., 1989; Akrawi et al., 1996) we can conclude that a slow system emerging in the long-range coupling via a slowing of axonal conduction velocity is a possible route to BS.

Our attempts to account for the dynamical pathogenesis of burst suppression differ from other approaches, most notably Ching et al. (2012). Ching et al. described the scalp EEG in terms of the activity of a small scale, biophysically detailed, computational model of interacting populations of cortical and thalamic neurons. Burst suppression was modeled as arising from metabolically induced alterations in an ATP-gated slow neuronal membrane potassium current $\left(I_{K_{ATP}}\right)$ on the basis that the reduction in cerebral metabolic rate (CMRO2) induced by anesthetic agents and hypoxia was associated with the depletion of ATP, and hence membrane hyperpolarization. While on this basis they claim to have accounted for a number of defining features of BS that included (i) the spatial synchrony of burst onset (ii) the parametric variability of burst duration/isoelectricity and (iii) the characteristically long time scales associated with bursting/isoelectricity compared to resting EEG, some caution needs to be exercised.

Firstly their model of resting/spontaneous EEG is constructed on the basis of the activity of no more than 20 model neurons. Because EEG is a distributed large scale phenomena such a model is unlikely to meaningfully account for resting/spontaneous activity particularly given the absence of any long-range excitatory cortico-cortical coupling. This has important implications for the propagation of burst activity particularly given that the onset of bursts, when examined at fine temporal scales, is probably not truly spatially homogeneous.

Secondly while the relationship between CMRO2 and ATP production cannot be reasonably disputed, not all anesthetic agents that produce reductions in CMRO2 produce BS. For example the noble gas xenon has been reported to reduce CMRO2 by up to 33% in human participants (Rex et al., 2006) yet is not associated with any discernable BS.

Thirdly the approach they have taken to producing BS essentially depends on the slow modulation of a faster system, the approach we have adopted here. The modeled time scales of $I_{K_{ATP}}$ variability are very long, of the order of tens of seconds.

For clarity and tractability the current investigations have focused on the spatially homogeneous case for the model of Liley et al. [i.e., ▽^2^ = 0 in equation (6)]. Clearly the emergence of BS in the spatially extended case will need to be investigated through the appropriate numerical solution of the defining partial differential equations. Because the cortical phase synchrony (Hartikainen et al., 1995) of burst suppression has not, as far as we are aware, been explicitly investigated it will be crucial to empirically determine the spatiotemporal emergence of bursts in order to assess the importance of excitatory cortico-cortical connectivity in the emergence and modulation of BS as implied by the model and as we have suggested. This will require recording high density EEG during anesthesia in which BS is present.

## Conflict of Interest Statement

The authors declare that the research was conducted in the absence of any commercial or financial relationships that could be construed as a potential conflict of interest.
